# 

**Published:** 1878-11

**Authors:** 


### Chicago Medical Journal

AND

### EXAMINER.

Vol. XXXVII.—NOVEMBER, 1878.—No. 5.

### To our Readers and Correspondents.

Beginning with Vol. XXXVII., July, 1878, the Chicago Medical Journal and Examiner prints all records of length and weight in terms of the Metric System, and all records of temperature in degrees of the Centigrade Scale. The Metric System, legalized in the United States and Great Britain, is extensively or exclusively employed by other civilized nations, and has thus become an essential part of the international language of science. It is recommended for adoption to the profession in this country by the American Medical Association and other scientific bodies. To-day no physician can afford to be ignorant of its value, its simplicity and the meaning of its terms.

The subjoined tables and scales, which have been kindly prepared for us by Prof. W. S. Haines, will be continuously reproduced in subsequent numbers of this journal, for the ready reference of our readers and correspondents.

### Metric Measures of Length.

| | | | | |
|---|---|---|---|---|
| Millimeter | 0.001 | of a Meter | 0.03937 | inches. |
| Centimeter | 0.01 | " " | 0.39370 | " |
| Decimeter | 0.1 | " " | 3.93707 | " |
| **Meter** | **1.** | **Meter** | **39.37079** | " |
| Decameter | 10. | Meters | 393.70790 | " |
| Hectometer | 100. | " | 3937.07900 | " |
| Kilometer | 1000. | " | 39370.79000 | " |

### Metrical Weights.

| | | | | |
|---|---|---|---|---|
| Milligram | 0.001 | of a Gram | 0.015 | grains. |
| Centigram | 0.01 | " " | 0.154 | " |
| Decigram | 0.1 | " " | 1.543 | " |
| **Gram** | **1.** | **Gram** | **15.432** | " |
| Decagram | 10. | Grams | 154.323 | " |
| Hectogram | 100. | " | 1543.234 | " |
| Kilogram | 1000. | " | 15434.348 | " |

☞ The United States "nickel" five cent piece weighs five grams, and is two centimeters in diameter.

### Approximate Equivalent of Metrical Weights.

*For Rapid Reference.*

A Kilogram=$2^{1}/_{5}$ lbs. Avoirdupois.

* The decimal *line* instead of points makes errors impossible.

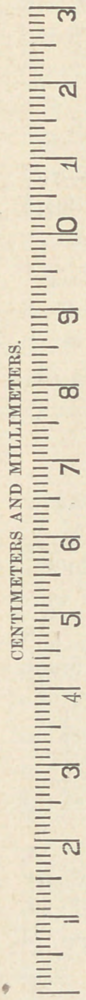

### Metric Fluid Measures.

When using the metric system, fluids are preferably prescribed by weight, employing the gram, its multiples and subdivisions, just the same as with solids, thus avoiding the errors due to refraction, adhesion, and inaccurate measuring vessels. For practical purposes four grams of water may be regarded as equivalent to a fluid drachm of that liquid, and the same may be considered true of tinctures and infusions; syrups, on the average, are about one-third heavier than water, so that a fluid ounce of a syrup will be approximately represented by 43 grams.

If preferred, however, fluids may be prescribed by volume in the metric, just as in the present system, using for that purpose the *Cubic Centimeter*, that is, a volume represented by a cube all of whose sides measure one centimeter. An ordinary back-gammon die is usually about this size. One cubic centimeter (written 1 C. C.) = 16.231 minims. It is approximately regarded as one fourth of a fluid drachm.

### Approximate Equivalents of Cubic Centimeter.

| | | | | |
|---|---|---|---|---|
| 0.001 | C. C. | = ......... | 1/65 | minim. |
| 0.01 | " | = ......... | 1/6 | " |
| 0.1 | " | = ......... | 1 1/2 | " |
| **1.** | **C. C.** | **= .................** | **15** | **minim.** |
| 4. | " | = ......... | 1 | fluid drachm. |
| 16. | " | = ......... | 4 | fluid drachms. |
| 32. | " | = ......... | 1 | ounce. |

1000 C. C. (usually known as a **Liter**) is a trifle more than one quart, wine measure.

The following prescription—

R: Potassii bromidi, ℥i.

Elixir aurantii, fl., ℥viij.

M.

Would, in metric terms, be written:

Potassic bromide, 32 |

Orange elixir, 250 |

### BOOKS AND PAMPHLETS RECEIVED.

A Treatise on the Science and Practice of Midwifery. By W. S. Playfair, M. D., F. R. C. P., etc., etc., with Notes and Additions by Robert P. Harris, M. D. Second American edition, from the second and revised London edition, with two plates and one hundred and eighty-two illustrations. Leather, pp. 638. 1878. Phil.: H. C. Lea. Chicago: Jansen, McClurg & Co.

The Antagonism of Therapeutic Agents: and what it Teaches. The Essay to which was awarded the Fothergillian gold medal of the Medical Society of London, for 1878. By T. J. Milner Fothergill, M. D., Edin. Cl. pp. 156. Phil.: H. C. Lea. Chicago: Jansen, McClurg & Co-

The Throat and its Diseases. With One Hundred Typical Illustrations in colors, and Fifty Wood Engravings, designed and executed by the author, Lenox Browne, F. R. C. S. Cloth, pp. 25. Philadelphia, H. C Lea. Chicago: Jansen, McClurg & Co.

A Treatise on the Diseases of the Ear, including the Anatomy of the Organ. By D. B. St. John Roosa, M. A., M. D. Fourth Edition. Illustrated by wood engravings and chromo-lithographs. Cloth, pp. 569. New York: Wood & Co. Chicago: Jansen, McClurg & Co.

Cyclopedia of the practice of Medicine. Edited by Dr. H. VonZiemssen. Vol. VIII. Diseases of the Chylopoetic System, with Chapters relating to Disease of the Bladder and Urethra and Functional Affections of the Male Genital Organs. Editor of American Edition, Albert H. Buck, New York. Cloth, pp. 935. 1878. New York: Wm. Wood & Co. Chicago: W. T. Keener, 65 Washington street.

Sore Throat, its Nature, Varieties and Treatment; Including the Connection between the Affections of the Throat and other Diseases. By Rosser James, M. D., M. R. C. P. Third edition, with hand colored plates. Cloth, pp. 288. 1878. Philadelphia: Lindsay & Blakiston. Chicago: Jansen, McClurg & Co.

A Monograph on the Treatment of Diphtheria, Based upon a New Etiology and Pathology. By Wm. C. Reiter, A. M., M. D. Cloth, pp. 47. 1878. Philadelphia: J. B. Lippincott & Co. Chicago: Jansen, McClurg & Co.

The Cell Doctrine, its History and Present State; for the use of Students in Medicine and Dentistry. Also a Copious Bibliograph on the Subject. By James Tyson, M. D. Second edition, revised and enlarged. Illustrated. Cloth, pp. 202. 1878. Philadelphia: Lindsay & Blakiston. Chicago: Janson, McClurg & Co.

A Guide to the Practical Examination of Urine, for the use of Physicians and Students. By James Tyson, M. D. Second edition, revised and improved, with illustrations. Cloth, pp. 172. 1878. Philadelphia: Lindsay & Blakiston. Chicago: Jansen, McClurg & Co.

Varicocele; Inaugural Dissertation zur Erlangung der Doctor Würde in der Medicin, Chirurgie und Geburtshilfe. Von Nicholaus Senn, aus Milwaukee, Wisconsin.

Placentarreste nach Zeitgeburten. Inaugural Disrertation zur Erlangung der Doctor Würde, in der Medicin, Chirurgie und Geburtshilfe. Von Alois Graettinger, aus Milwaukee, Wis.

Thirty-Fifth Annual Report of the Managers of the State Lunatic Asylum, Utica, N. Y., for the year 1877.

The Fifty-Fourth Annual Report of the Officers of the Retreat for the Insane at Hartford, Connecticut. April, 1878.

Fifteenth Annual Report of the New York Society for the Relief of the Ruptured and Crippled. May, 1878.

Upon the Treatment of Strumous Diseases by what we may call the Solfatara Method. By H. R. Storer, M. D. Reprint from the Boston Medical and Surgical Journal. June 27, 1878.

Strictures of the Cervical Canal. By Fred. Ekland, M. D. Translated from the Swedish, by A. Sibley Campbell, A. B., M. D., Augusta, Georgia.

Operations of Closure of Cleft Palate. By A. Vanderveer, M. D. One of the Series of American Clinical Lectures. Edited by E. C. Seguin.

Report of Public Hygiene in Indiana. By Thad. M. Stevens, M. D., Indianapolis.

Restriction and Prevention of Diphtheria. Document issued by the State Board of Health of Michigan.

On the use of the Solid Rubber Bandage in the Treatment of Eczema and Ulcers of the Leg. By L. Duncan Bulkley, A. M., M. D. Reprint from the Archives of Dermatology. July, 1878.

On Diet and Hygiene in Diseases of the Skin. By L. Duncan Bulkley, A. M., M. D. Reprint from Virg. Med. Monthly.

Elettromopatia. By Conte Cesare Matthei.

Emiplegia, Faringo-Laringea Destra. By Giovanni Loughi.

La Scienza Nuova del Conte Cesare Matthei et la Scienza Vecchia del Dottore C.

---

MEDICAL QUACKS IN BAVARIA.—Since 1873, an official census is taken in Bavaria every year, of all persons who are practicing medicine or surgery without having passed the examination. These people are allowed to practice under certain restrictions which do not seem to discourage them at all, for the regular physicians begin to get alarmed by the rapid increase of quackery. In a recent number of the *Ærztliche Intelligenz-Blatt* the statistics are given for the past four years, which show that the number of quacks in Bavaria has increased from 1,156 in 1874 to 1,563 in 1877. The quacks are of all kinds; but, prominent among them are the barbers, midwives, druggists, and ministers of the Gospel.

### ANNOUNCEMENTS FOR THE MONTH.

### SOCIETY MEETINGS.

Chicago Medical Society—Mondays, Nov. 4 and 18.
West Chicago Medical Society—Mondays, Nov. 11 and 25.

### CLINICS.

MONDAY.

Eye and Ear Infirmary—2 p. m., Ophthalmological, by Prof. Holmes; 3 p. m., Otological, by Prof. Jones.

Mercy Hospital—1:30 p. m., Surgical, by Prof. Andrews.

Rush Medical College—2 p. m., Dermatological and Venereal, by Dr. Hyde; 3 p. m., Medical, by Dr. Bridge.

Woman's Medical College—2 p. m., Dermatological, by Dr. Maynard.

TUESDAY.

Mercy Hospital—1:30 p. m., Medical, by Prof. Hollister.

WEDNESDAY.

Chicago Medical College—1:30 p. m., Eye and Ear, by Prof. Jones.

Eye and Ear Infirmary—2 p. m., Ophthalmological, by Dr. Hotz.

THURSDAY.

Chicago Medical College—1:30 p. m., Medical, by Prof. Quine.

FRIDAY.

Mercy Hospital—1:30 p. m., Medical, by Prof. Davis.

SATURDAY.

Rush Medical College—2 p. m., Surgical, by Prof. Gunn.

Chicago Medical College—2 p. m., Surgical, by Prof. Isham; 3 p. m., Neurological, by Prof. Jewell.

Woman's Medical College—11 a. m., Ophthalmological, by Dr. Montgomery.

Daily Clinics, from 2 to 4 p. m., at the Central Free Dispensary, and at the South Side Dispensary.

